# Mapping Guaranteed Positive Secret Key Rates for Continuous Variable Quantum Key Distribution

**DOI:** 10.3390/e26100865

**Published:** 2024-10-15

**Authors:** Mikhael T. Sayat, Oliver Thearle, Biveen Shajilal, Sebastian P. Kish, Ping Koy Lam, Nicholas J. Rattenbury, John E. Cater

**Affiliations:** 1 Department of Physics, Faculty of Science, University of Auckland, Auckland 1010, New Zealand; 2 Department of Quantum Science and Technology, Research School of Physics, Australian National University, Canberra 2601, Australia; 3 Quantum Innovation Centre (Q.InC), Agency for Science Technology and Research (A*STAR), 2 Fusionopolis Way, Innovis #08-03, Singapore 138634, Singapore; 4 Institute of Materials Research and Engineering (IMRE), Agency for Science Technology and Research (A*STAR), 2 Fusionopolis Way, Innovis #08-03, Singapore 138634, Singapore; 5 Sensors and Effectors Division, Defence Science and Technology Group, Adelaide 5111, Australia; 6 Data61, Commonwealth Scientific and Industrial Research Organisation, Sydney 2015, Australia; 7 Department of Mechanical Engineering, University of Canterbury, Christchurch 8041, New Zealand

**Keywords:** quantum key distribution, continuous variable, discrete modulated, protocol, comparison tool, numerical analysis

## Abstract

The standard way to measure the performance of existing continuous variable quantum key distribution (CVQKD) protocols is by using the achievable secret key rate (SKR) with respect to one parameter while keeping all other parameters constant. However, this atomistic method requires many individual parameter analyses while overlooking the co-dependence of other parameters. In this work, a numerical tool is developed for comparing different CVQKD protocols while taking into account the simultaneous effects of multiple CVQKD parameters on the capability of protocols to produce positive SKRs. Using the transmittance, excess noise, and modulation amplitude parameter space, regions of positive SKR are identified to compare three discrete modulated (DM) CVQKD protocols. The results show that the *M*-QAM protocol outperforms the *M*-APSK and *M*-PSK protocols and that there is a non-linear increase in the capability to produce positive SKRs as the number of coherent states used for a protocol increases. The tool developed is beneficial for choosing the optimum protocol in unstable channels, such as free space, where the transmittance and excess noise fluctuate, providing a more holistic assessment of a protocol’s capability to produce positive SKRs.

## 1. Introduction

Quantum key distribution (QKD) is the sharing of keys between communicating parties, Alice and Bob, where the presence of an eavesdropper, Eve, can be inferred as a consequence of fundamental quantum mechanics [1,2]. Continuous variable QKD (CVQKD) uses the phase and amplitude of a laser to generate a secret key between Alice and Bob [3,4].

The governing performance metric in CVQKD is the secret key rate (SKR), which measures the rate at which a secret key can be generated between communicating parties [5,6]. The SKR is calculated based on varying an independent variable while keeping all other parameters constant. This can be seen for the Gaussian-modulated (GM) CVQKD protocol where the SKR is calculated based on parameters such as the reconciliation efficiency, excess noise, and link distance [5,7]. The same method is applied for the discrete modulated (DM) CVQKD protocols such as the *M*-QAM [6], *M*-APSK [8,9], and *M*-PSK [10,11,12,13] protocols. The same is observed in the analysis and feasibility studies of different CVQKD protocols in different free-space link types such as inter-satellite [14,15,16,17], satellite-to-ground [18,19,20,21,22], underwater link [23,24,25], and fibre [26,27,28,29] links. By keeping all but one parameter constant in the calculation of the SKR, the simultaneous effects of parameters can be missed and/or overlooked, and the optimum protocol for a given channel can be difficult to determine. This is especially crucial in free-space channels where parameters such as the channel transmittance and excess noise fluctuate [5,18]. It may then be beneficial to study parameter ranges which allow positive SKRs to account for fluctuating parameters.

In this work, for the first time, a numerical tool is developed for comparing different CVQKD protocols for different channels and identifying regions of positive SKR in the transmittance–excess noise–modulation amplitude parameter space. The three parameters that were chosen as practical CVQKD systems can form a feedback loop whereby the modulation amplitude can be tuned to the potentially fluctuating transmittance and excess noise in the channel to maximise the SKR. The tool can then be used to identify the appropriate modulation amplitude values for the estimated transmittance and excess noise ranges due to fluctuations. To showcase the capabilities of the tool, the *M*-QAM, *M*-APSK, and *M*-PSK DM-CVQKD protocols are compared.

## 2. DM-CVQKD Protocols

In CVQKD, information is encoded in the two quadratures of light by modulating coherent states in the continuous amplitude (*x*) and phase quadratures (*p*). A series of coherent states, each one represented by the notation |α〉=|x+ip〉, are sent from Alice to Bob where they are measured. If Eve was to intercept any of the transmitted states, then their presence would appear as noise and loss to Alice and Bob due to the no cloning theorem [7]. The distribution of these coherent states on the phase space is discrete for DM-CVQKD.

The SKR is the lower bound on the key rate calculated by Alice and Bob by determining their shared information ($I_{AB}$) and subtracting the upper bound of the inferred information intercepted by Eve ($S_{BE}$). The SKR is calculated as

(1) $$\mathrm{SKR}=\beta I_{AB}-S_{BE},$$

where β is the reconciliation efficiency, a method of error correction on the transmitted and received coherent states [5].

The parameters and SKR can be calculated in two ways. The first method uses a linear channel assuming (LCA) security analysis [11,30,31]. The second uses a semi-definite programming (SDP) method [6,12,31,32] that requires more computational effort but has a more general secure analysis theory than the LCA method.

In developing the tool, the calculation of the SKR for the three DM-CVQKD protocols employs the analytical LCA method from Denys et al. [6] which is secure against collective attacks. Collective attacks are a form of attack where Eve uses ancilla states for an independent and identically distributed attack, stores the state in a quantum memory, and later performs an optimal collective measurement [5]. Although optimal attacks are unknown for DM-CVQKD, collective attacks are usually optimal in the asymptotic limit [6,8]. Using the de Finetti theorem and the global representation theorem for symmetric states, QKD protocols that are secure against collective attacks imply security against general and arbitrary attacks [33,34]. As a universal finite size limit, SKR security proofing for DM-CVQKD is still an active field of research [6,35,36,37], and analysis has been restricted to the asymptotic limit. In addition, the focus of this work is the tool and not the underlying protocols and security proofs. The remainder of this section describes the distribution of coherent states on the phase space for *M*-PSK, *M*-QAM, and *M*-APSK.

### 2.1. *M*-PSK: Phase Shift Keying

**Figure 1:**
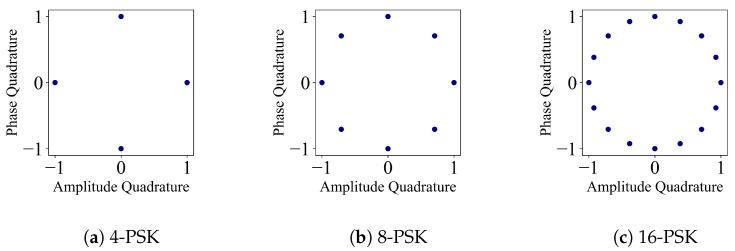
Phase space representation of 4, 8, 16-PSK protocols, α=1. Each coherent state has uniform probability $\left(\frac{1}{M}\right)$.

*M*-PSK is a distribution of *M*-modulated coherent states on the phase space with a constant value of α and a uniform probability $\left(\frac{1}{M}\right)$ [11,13], and can be depicted on the phase space as shown in Figure 1. A modulated coherent state takes the form

(2) $$|\alpha_{k}\rangle =\alpha \exp\left(\frac{i2\pi k}{M}\right).$$

### 2.2. *M*-QAM: Quadrature Amplitude Modulation

*M*-QAM is a distribution of *M* modulated coherent states on the phase space following a grid-like pattern where each coherent state is assigned a different probability [6].

A modulated coherent state takes the form

(3) $$\begin{array}{c} |\alpha_{k,l}\rangle =\frac{\alpha \sqrt{2}}{\sqrt{m-1}}\left(k-\frac{m-1}{2}\right)+i\frac{\alpha \sqrt{2}}{\sqrt{m-1}}\left(l-\frac{m-1}{2}\right), \end{array}$$

where $M=m^{2}$, and the coherent states are equidistantly spaced between $-\sqrt{m-1}$ and $\sqrt{m-1}$ in the phase and amplitude quadratures. Here, k,l∈{0,1,…,(m−1)}. The associated probability for each each coherent state, $p_{k,l}$, can follow either a binomial distribution, as follows:

(4) pk,l=122(m−1)m−1km−1l,

or a discrete Gaussian distribution, as follows:

(5) $$\begin{array}{c} p_{k,l}=\exp\left(-v\left(x^{2}+p^{2}\right)\right), \end{array}$$

where $x=\frac{\alpha \sqrt{2}}{\sqrt{m-1}}\left(k-\frac{m-1}{2}\right)$ and $p=\frac{\alpha \sqrt{2}}{\sqrt{m-1}}\left(l-\frac{m-1}{2}\right)$.

**Figure 2:**
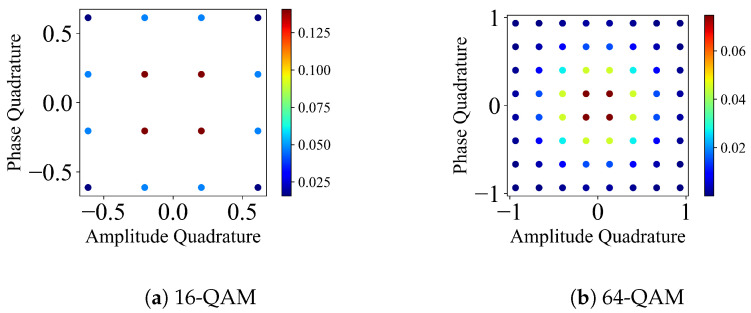

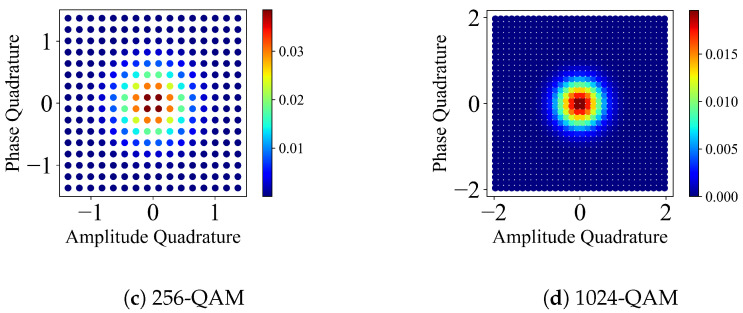
Phase space representation of 16, 64, 256, 1024-QAM protocols, α=0.5. Colour bars indicate the probability of the coherent state for the particular protocol.

*M*-QAM on the phase space is depicted as shown in Figure 2.

### 2.3. *M*-APSK: Amplitude Phase Shift Keying

*M*-APSK is a distribution of *M*-modulated coherent states on the phase space where the coherent states are placed in concentric rings [8,9]. Each ring has a particular number of coherent states. A modulated coherent state takes the form

(6) $$|\alpha \rangle =\beta_{p}\alpha \exp\left(\frac{i2\pi k}{M_{p}}\right),$$

where $\beta_{p}=\frac{1}{R},\frac{2}{R},\ldots ,1$ (*R* is the number of rings), $M_{p}$ is the number of coherent states in a ring (4, 12, 16, 32, 64, 128, 256 from 1st to 7th ring), and p=1,2,…,R.

**Figure 3:**
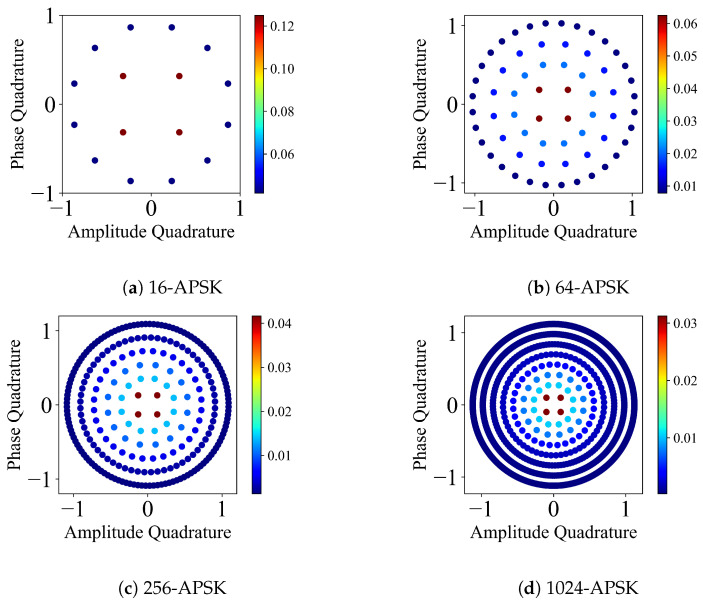
Phase space representation of 16, 64, 256, 1024-APSK protocols, α=0.707. Colour bars indicate the probability of the coherent state for the particular protocol.

A coherent state in a particular ring has equal probability $\left(\frac{1}{M_{p}}\right)$, and each ring has the same probability $\left(\frac{1}{R}\right)$. Therefore, each coherent state has a probability of $\frac{1}{RM_{p}}$ in the case of a discrete uniform distribution. *M*-APSK can also have non-uniform distributions [8,9]. *M*-APSK on the phase space is depicted in Figure 3.

## 3. Methods

**Figure 4:**
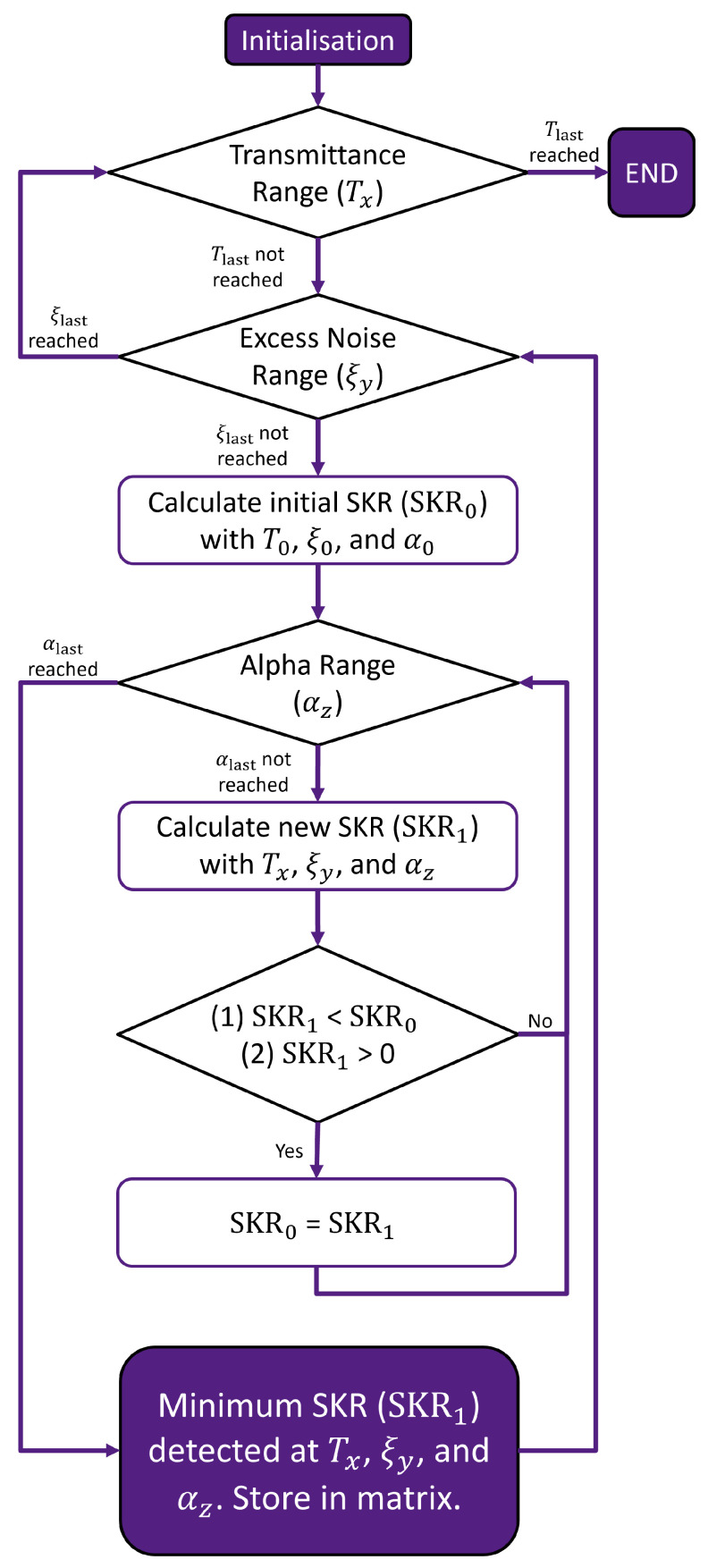
Process for calculating the minimum positive SKR of a protocol for a given *T*, ξ, and α. The minimum positive SKR values create the minimum positive SKR boundary from which the level of the boundary can be calculated and used to compare different protocols.

A boundary surface can be determined to identify regions of positive and negative SKR. In addition, the shape of the surface provides an indication of how different CVQKD parameters simultaneously affect the capability of a protocol to produce positive SKRs in different channels. To identify the boundary, a grid search of CVQKD parameters is performed (Figure 4). In this case, the CVQKD parameters considered are the transmittance (*T*), excess noise (ξ), and the modulation amplitude (α) (which is related to the modulation variance by $V_{A}=2\alpha$). Therefore, the boundary for a 3D comparative analysis exists in the T−ξ−α parameter space.

For specified values of *T* and ξ, an initial SKR is calculated using a value of α within a defined range. For each value within the α range, the minimum positive SKR is found iteratively by calculating a new positive SKR and comparing it to the initial SKR, or the previous minimum positive SKR. A new minimum positive SKR is found by satisfying the inequality:

(7) $$\begin{array}{c} 0<{\mathrm{SKR}}_{1}<{\mathrm{SKR}}_{0}, \end{array}$$

where ${\mathrm{SKR}}_{1}$ is the new SKR within the α range, and ${\mathrm{SKR}}_{0}$ is the previous minimum positive SKR or the initial SKR value during the first iteration. Once the final α value within the α range is reached, the current minimum positive SKR for specific *T*, ξ, and α is stored in a matrix. The process is complete when a minimum positive SKR is found for each combination of *T*, ξ, and α. A three-dimensional surface can then be fitted with a polynomial.

**Figure 5:**
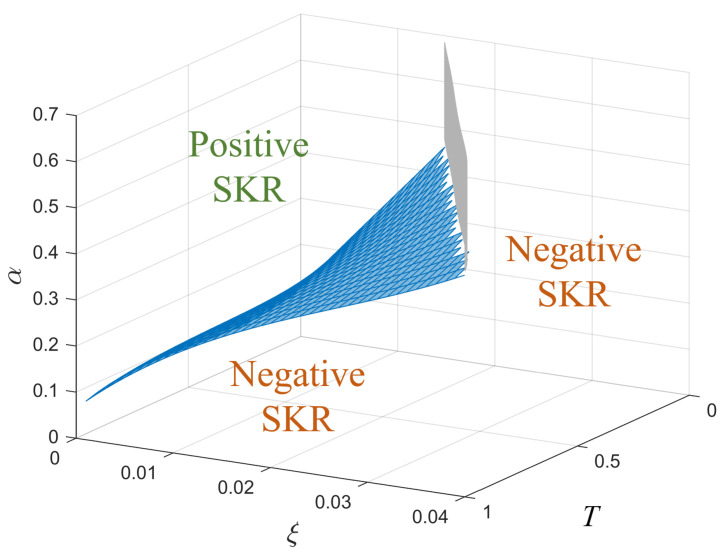
The boundary for 16-APSK (blue). The cut-off plane (grey) is shown to separate regions of positive SKR and negative SKR in three dimensions.

Figure 5 shows the discretised mesh with a surface fit representing the boundary for 16-APSK, identifying the regions of positive and negative SKR. Here, a protocol’s boundary shown in blue (in this case for 16-APSK) corresponds to the specific *T*, ξ, and α values which yield an SKR of 0, and allows the identification of regions of positive and negative SKR in the *T*–ξ–α parameter space. The vertical grey cut-off on the T−ξ plane shown is protocol-dependent and based on the defined α range (in this case, 0.1≤α≤0.5). If the α range was changed, then the position of the cut-off would also change.

The metric for comparing the capability to produce positive SKRs for different protocols is defined as the level of the boundary on the three-dimensional *T*–ξ–α parameter space (Figure 5). The level is defined as the average α value, $\alpha_{\mathrm{ave}}$, of the discretised boundary from the matrix when the gird search is complete. A protocol with a boundary with a lower level (smaller $\alpha_{\mathrm{ave}}$) has a larger capability of producing positive SKRs as there is a larger α range above the boundary that can produce positive SKRs [11]. This stems from protocols having a constrained modulation amplitude range in which positive SKRs can be produced. In this case, the 3D boundary generated from the grid search represents the modulation amplitude lower limit while taking into account the simultaneous effects of transmittance and excess noise.

It can be seen in Figures 3 and 5 in Ref. [11] that the modulation variance (proportional to the modulation amplitude) has a lower and upper limit depending on the channel, shown as when the SKR tends to 0. The tool’s boundary level represents the the lower limit in 3D. A boundary with a smaller $\alpha_{\mathrm{ave}}$ which corresponds to a smaller modulation variance/amplitude lower limit has a larger modulation variance/amplitude upper limit and hence a larger α range to produce positive SKRs. Conversely, a boundary with a larger $\alpha_{\mathrm{ave}}$ which corresponds to a larger modulation variance/amplitude lower limit has a smaller modulation variance/amplitude upper limit and hence a smaller α range to produce positive SKRs. The boundary level defined by $\alpha_{\mathrm{ave}}$ can then be used as a metric to determine the capability of a protocol to produce positive SKRs.

## 4. Results

The boundaries for the *M*-PSK, *M*-QAM, and *M*-APSK protocols were determined for comparison. The asymptotic limit SKR was used for the analysis in combination with the parameters in Table 1. Although the graphs are in 3D, 2D snapshots are shown to best display the shape of the boundaries.

The surface fit for the discretised mesh is a 3rd-order polynomial in three dimensions that can be expressed as

(8) $$\begin{array}{c} \alpha =c_{1}+c_{2}T+c_{3}\xi +c_{4}T^{2}+c_{5}T\xi +c_{6}\xi^{2}+c_{7}T^{3}+c_{8}T^{2}\xi +c_{9}T\xi^{2}+c_{10}\xi^{3}, \end{array}$$

where *T* is the transmittance and ξ is the excess noise. The coefficients of the surface fit ($c_{i}$) are protocol-dependent and can be further used in the calculation of $\alpha_{\mathrm{ave}}$ for a given *T* and ξ range. The surface fit can then be used for protocol comparisons. The coefficients for the surface fits were calculated for 16-PSK, 16-QAM, 16-APSK, 64-APSK, and 256-APSK (Table 2) for an intra-protocol comparison (different numbers of coherent states) and inter-protocol comparison (different modulation schemes). The R-squared value was included as a goodness of fit measure. For different protocols, the coefficients had to be re-calculated to determine the surface fit of the discretised mesh of a protocol. It should be noted that the surface fit (Equation (8)) is only shown for a visual representation on the *T*–ξ–α parameter space for a visual comparison and can be changed. The quantitative comparison arises from the comparison of $\alpha_{\mathrm{ave}}$. The similarity of the different protocols in small excess noise regimes was also studied and can be found in Appendix A.

### 4.1. Intra-Protocol Comparison

**Figure 6:**
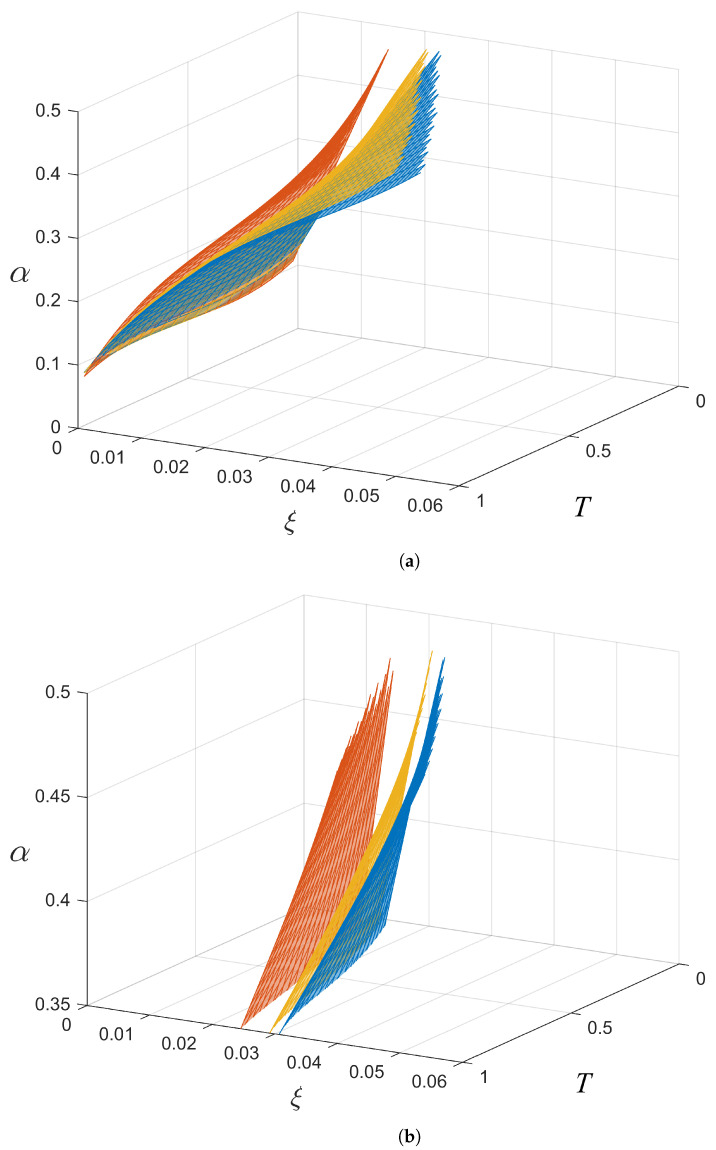
(**a**) Boundaries for 16-APSK (red), 64-APSK (yellow), and 256-APSK (blue). A closeup of the boundaries in part (**b**) shows that the gap between 16-APSK and 64-APSK is larger than the gap between 64-APSK and 256-APSK, showing that the level of the boundary non-linearly decreases as the number of coherent states (*M*) increases.

Figure 6 shows the effects of varying the number of coherent states for a given protocol; in this case, they show the effects for the *M*-APSK protocol. The coefficients, $c_{i}$, for the intra-protocol comparison are presented in Table 2. Increasing the number of coherent states lowers the level of the boundary and has the advantage of having positive SKRs for smaller *T* and larger ξ values. The larger difference in levels between 16 and APSK and 64-APSK, compared to 64-APSK and 256-APSK, shows that further increases in the number of coherent states for a protocol leads to incremental decreases in the level and therefore in incremental increases in the capability to produce positive SKRs.

For a fair comparison, the calculation of $\alpha_{\mathrm{ave}}$ was confined to ξ= 0–0.042 SNU and T= 0–1 as this is the region where 16-APSK, the worst performing of the three, produces positive SKRs. The results show that the $\alpha_{\mathrm{ave}}$ values of the boundaries for 16-APSK, 64-APSK, and 256-APSK are

- 16-APSK: $\alpha_{\mathrm{ave}}$ = 0.304 SNU.
- 64-APSK: $\alpha_{\mathrm{ave}}$ = 0.273 SNU.
- 256-APSK: $\alpha_{\mathrm{ave}}$ = 0.265 SNU.

**Figure 7:**
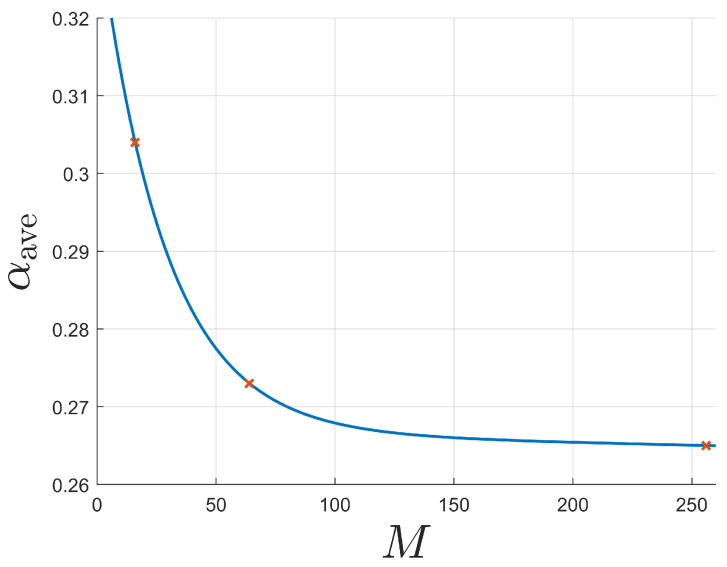
Non-linear decrease in the level of the boundary, $\alpha_{\mathrm{ave}}$, as the number of coherent states, *M*, increases. Shown for *M*-APSK.

It is observed that $\alpha_{\mathrm{ave}}$ decreases as the number of coherent states increases. In addition, the decrease in $\alpha_{\mathrm{ave}}$ from 64-APSK to 256-APSK is smaller than from 16-APSK to 64-APSK. This is consistent with the non-linear decrease in the level of the boundary as the number of coherent states further increases, as shown in Figure 7.

The protocol which has the largest capability to produce positive SKRs is 256-APSK as it has a larger α range above its boundary to produce positive SKRs. It has the smallest value of $\alpha_{\mathrm{ave}}$ and can produce positive SKRs for larger values of ξ and lower values of *T*.

### 4.2. Inter-Protocol Comparison

**Figure 8:**
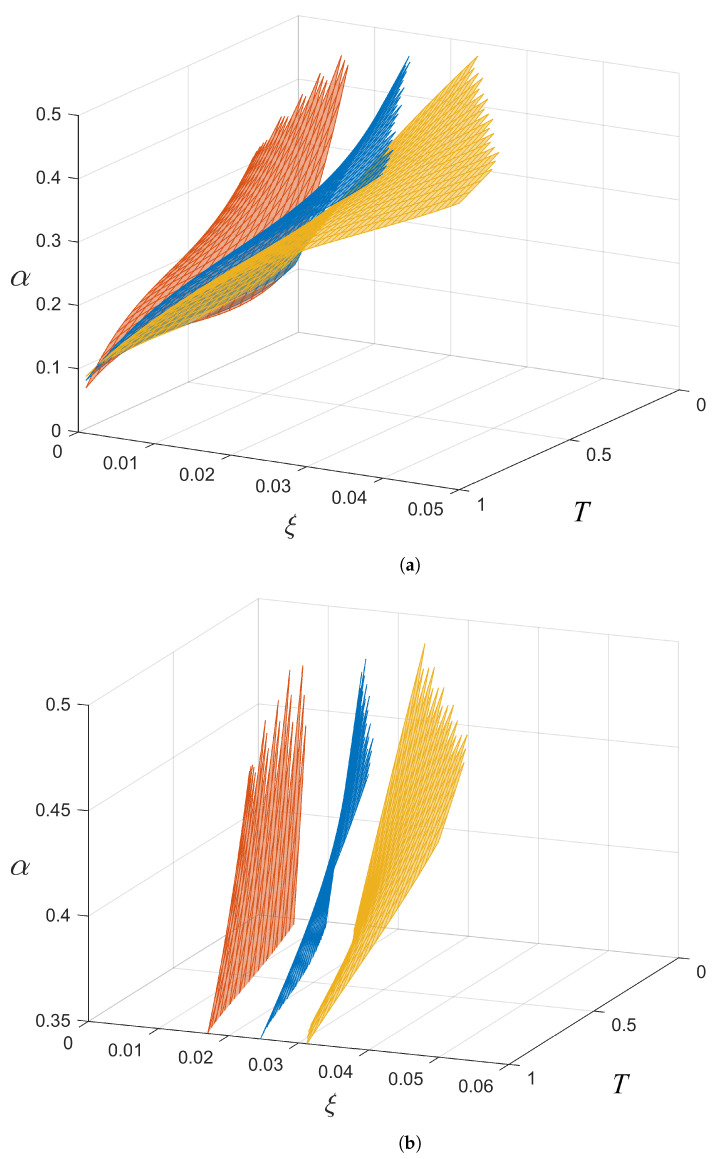
(**a**) Boundaries for 16-PSK (red), 16-APSK (blue), and 16-QAM (yellow). It can be seen that *M*-QAM is more resilient to unfavourable channel parameters (smaller *T* and larger ξ) as it has a lower level boundary. A closeup of the boundaries in part (**b**) shows that *M*-QAM can produce positive SKRs at higher levels of ξ.

Figure 8 shows the effects of changing protocols with the same number of coherent states; in this case, it shows the effects for the 16-PSK, 16-APSK, and 16-QAM protocols. The coefficients, $c_{i}$, for the inter-protocol comparison are presented in Table 2.

In this case, the calculation of $\alpha_{\mathrm{ave}}$ was confined to ξ= 0–0.023 SNU and T= 0–1 as this is the region where 16-PSK, the worst performing of the three, produces positive SKRs. The results show that the $\alpha_{\mathrm{ave}}$ values of the boundaries for 16-PSK, 16-APSK, and 16-QAM are

- 16-PSK: $\alpha_{\mathrm{ave}}$ = 0.256 SNU.
- 16-APSK: $\alpha_{\mathrm{ave}}$ = 0.210 SNU.
- 16-QAM: $\alpha_{\mathrm{ave}}$ = 0.193 SNU.

The 16-QAM protocol has the largest capability to produce positive SKRs compared to both 16-PSK and 16-APSK by having the smallest $\alpha_{\mathrm{ave}}$. In addition, the 16-QAM boundary extends to smaller *T* and larger ξ values, meaning that it has greater resilience in more adverse channels compared to the other protocols. In comparison to Figure 6, it can be seen in Figure 8 that the distances between the boundaries for the different protocols are larger. This is evidence that some protocols perform better than others (in this case, *M*-QAM is the best), and that changing protocols may be better (performance-wise) than increasing the number of coherent states in a protocol for a given *T* and ξ range.

## 5. Discussion

Within the many available link types for CVQKD, the transmittance and excess noise for a given channel may vary due to the sensitivity of the channel to its immediate environment. For example, crosstalk and Raman scattering in a fibre link could increase the noise in the channel and therefore cause the SKR to decrease [5]. In a free-space link, strong turbulence and a high concentration of aerosols would decrease the transmittance and therefore the SKR [22]. In 2D representations, the dependency of the SKR is only for one parameter. For example, the SKR depends on the transmittance (or excess noise) while keeping other parameters fixed. However, this restricts analyses and comparisons by omitting the simultaneous influence of other CVQKD parameters. Higher dimension analyses in larger parameter spaces to include the effects of as many parameters as possible can therefore be used. This is to make sure that for a given varying channel, the correct protocol can be chosen/implemented where adjustments in the modulation variance/amplitude would still guarantee positive SKRs.

The use of boundaries and $\alpha_{\mathrm{ave}}$ as metrics for comparing different protocols relies on the symmetric behaviour of SKRs as a function of the modulation variance/amplitude. This foundation can be extended to a 3D volume for a more holistic comparison. In this case, the volume would be enclosed by the boundaries corresponding to the lower and upper modulation variance/amplitude limits, the cut-off, and the CVQKD parameter ranges being studied. The parameter space within the volume would correspond to positive SKRs, including the parameters that result in high SKRs and the maximum positive SKR achievable. Conversely, the space outside the volume would correspond to negative SKRs. As a result, the capability of a protocol to produce positive SKRs depends on the enclosed volume shape and size.

Comparisons for different existing and future CVQKD protocols should include all CVQKD parameters (related to the channel and technology available) for an overall comparison. This would lead to an *n*-dimensional comparative analysis where *n* represents the number of CVQKD parameters studied for comparing protocols. An immediate extension to the boundary surface or volume would be the incorporation of the SKR. As it is, the transmittance, excess noise, and modulation amplitude are studied to identify positive and negative SKR regions, where the SKR is used to determine the boundary but is not presented. The incorporation of the SKR in the T−ξ−α parameter space would provide a more comprehensive comparison as regions of larger and smaller positive SKRs can be identified. In a 3D volume, this would require slices within the volume with heat maps identifying larger and smaller regions of positive SKR.

Aside from the incorporation of the SKR, other CVQKD parameters (coupling and detector efficiencies [5], reconciliation efficiency [38,39,40], etc.) can be incorporated. Parameters inherent to different types of links and nodes could be included, e.g., aperture diameters [19,22], losses in Alice and Bob [41], chlorophyll concentrations (underwater links) [23,24,25], etc. These would again be incorporated as heat maps on a 3D volume, adding another dimension for comparison. Conversely, a mix of different CVQKD parameters could be used, presenting a new 3D parameter space, different from the T−ξ−α parameter space, for comparison.

## 6. Conclusions

A boundary surface representing the border between positive and negative SKRs was developed on a three-dimensional transmittance–excess noise–modulation amplitude parameter space. This representation can identify regions of positive and negative SKRs, avoiding the individual analysis of separate parameters with the SKRs. The boundary surface level as determined by the $\alpha_{ave}$ metric can be used to compare the capability of different CVQKD protocols (in this case, *M*-PSK, *M*-QAM, and *M*-APSK) to produce positive SKRs while taking into account simultaneous effects of different CVQKD parameters (in this case, the transmittance, excess noise, and modulation amplitude). A smaller $\alpha_{ave}$ represents a larger capability to produce positive SKRs. Conversely, a larger $\alpha_{ave}$ represents a smaller capability to produce positive SKRs. Using this metric, the *M*-QAM protocol outperforms the *M*-APSK protocol, which outperforms the *M*-PSK protocol when M=16. In addition, a larger number of coherent states (*M*) non-linearly increases the capability of a protocol to produce positive SKRs. The three-dimensional boundary surface representation can also identify regions where the performance of protocols are similar, in accordance with the previous two-dimensional representations, e.g., low excess noise regimes. The comparison of different protocols is essential for choosing the optimum protocol for a given channel. In real channel links where parameters differ and fluctuate, a comparison tool that identifies parameter ranges (regions) with a positive SKR is beneficial for choosing the optimum protocol for CVQKD to be feasible.

## Figures and Tables

**Table 1:** CVQKD parameters.

Parameter	Value
Transmittance (*T*)	0–1 (increments of 0.05)
Excess noise (ξ)	0.001–0.5 (increments of 0.001)
Alpha (α)	0.1–0.5 (increments of 0.0001)
Reconciliation Efficiency (β)	0.95
Detection	Heterodyne

**Table 2:** Numerical coefficients for the 16-PSK, 16-QAM, 16-APSK, 64-APSK, 256-APSK surface fits.

Coefficient	16-PSK	16-QAM	16-APSK	64-APSK	256-APSK
$c_{1}$	0.09639	0.1118	0.1007	0.1081	0.1114
$c_{2}$	–0.2603	–0.1984	–0.2023	–0.1963	–0.1970
$c_{3}$	36.92	19.61	26.09	21.39	19.85
$c_{4}$	0.6855	0.4022	0.4828	0.4083	0.4009
$c_{5}$	–62.34	–11.44	–24.24	–13.88	–11.75
$c_{6}$	1203	–193.4	–130.1	–204.8	–194.2
$c_{7}$	–0.4796	–0.2398	–0.3155	–0.246	–0.2396
$c_{8}$	56.30	5.976	16.24	7.638	6.253
$c_{9}$	–2614	–43.86	–250.8	–67.33	–49.49
$c_{10}$	38,670	2101	4708	2640	2195
R-Square	0.9822	0.9980	0.9976	0.9977	0.9978

**Figure A1:**
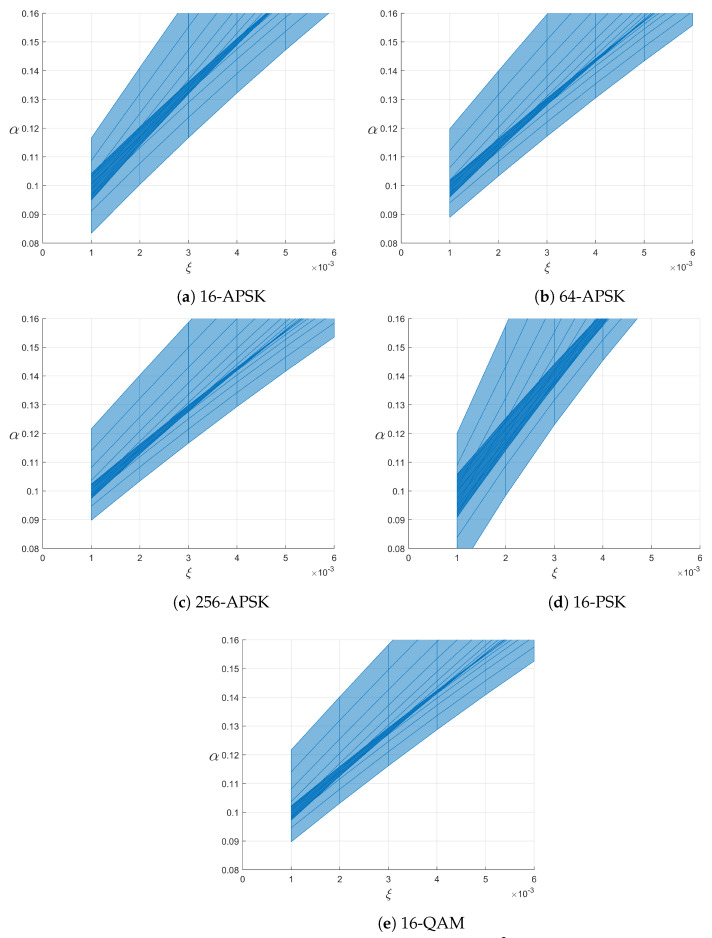
The boundary for small excess noise regimes ($\xi \;∼\;10^{-3}$) for the protocols studied. The similar levels of the boundaries, indicate similar capabilities for producing positive SKRs at small excess noise regimes. The boundary for small excess noise regimes.

## Data Availability

The original contributions presented in the study are included in the article, further inquiries can be directed to the corresponding author.

## Appendix A. Similar Regions

As shown in Figure 6 and Figure 8, the boundaries intersect in small excess noise regimes ($\xi \;∼\;10^{-3}$). A closeup of this regime is shown in Figure A1 for all transmittance values (T= 0–1). The overlapping boundaries mean that the capability of *M*-PSK, *M*-QAM, and *M*-APSK to produce positive SKRs are similar for all values of *M*.

In practical CVQKD implementations, different CVQKD protocols may vary in complexity. Three-dimensional comparisons can identify regimes where the requirements for guaranteeing a positive SKR are relaxed, such as in small excess noise regimes (Figure A1). In this case, the task of choosing a protocol is made redundant as they all have similar performances regardless of the number of coherent states or modulation scheme.
